# The silent accumulation: AI as mental contaminant

**DOI:** 10.3389/frai.2026.1755151

**Published:** 2026-04-02

**Authors:** Vladimír Šucha

**Affiliations:** European University Institute, Florence, Italy

**Keywords:** algorithmic impact assessment, artificial intelligence governance, cognitive environmental contamination, cognitive-social ecosystem services, cumulative exposure, human agency

## Abstract

This paper introduces a theoretical framework for understanding how seemingly innocuous AI systems create cumulative effects on human cognition, emotion, and agency that current governance approaches fail to address. Drawing on environmental health science, we propose that “low-risk” AI applications, those falling below regulatory thresholds in frameworks such as the EU AI Act, function as cognitive environmental contaminants whose collective and sustained presence may reshape human psychological capacities. We operationalise cumulative AI exposure along five dimensions (frequency, duration, intensity, diversity of systems, and developmental timing) and identify five pathways through which cumulative effects may manifest: attention erosion, emotional dependency, social connection alteration, decision-making dependency, and identity fragmentation. For each pathway, we distinguish empirical regularities documented in existing research, plausible mechanisms through which cumulative effects may operate, and speculative population-level hypotheses that require empirical testing. Situating our framework against adjacent literatures including technostress, cognitive offloading, hypernudging, and automation bias, we argue that the distinctive contribution lies in the cumulative, cross-system, population-level analytical paradigm and its governance translation. We propose three governance mechanisms—cumulative impact assessment extending existing algorithmic auditing frameworks, cognitive-social environmental monitoring using validated psychometric instruments, and economic valuation of cognitive-social ecosystem services, accompanied by a phased validation strategy. The framework is offered as a complement to existing risk-based governance, addressing a specific gap: the systematic invisibility of effects that emerge from the interaction of multiple AI systems over extended time periods.

## 1 Introduction

When environmental scientists first began measuring trace amounts of heavy metals in water supplies or atmospheric particulates in urban environments, these levels were often deemed “acceptable” or “low-risk” by regulatory standards. Individual exposures appeared inconsequential. Yet decades later, the cumulative impact of these persistent, low-level contaminants revealed devastating consequences: cognitive impairment in children exposed to lead, cardiovascular disease from air pollution, and bioaccumulation of toxins throughout ecosystems (UNEP, 2025).

We now face a parallel situation with artificial intelligence, not in our physical environment, but in our cognitive and social landscape. While governance frameworks focus primarily on high-risk AI applications, this paper explores how seemingly innocuous, “low-risk” AI systems may, through cumulative exposure, fundamentally reshape human agency and societal functioning in ways that current technological governance approaches fail to address.

This phenomenon is the complex interplay between technological development and social transformation, where the choices available to society regarding technology use have profound implications for human flourishing (Winner, 1980). As Bijker et al. (1987) demonstrated, technological artifacts are socially constructed, but they also construct social realities in return. Our framework extends this insight to show how AI’s social construction of reality operates through subtle, cumulative cognitive and emotional influences that accumulate below the threshold of regulatory attention.

This paper makes three primary contributions. First, we introduce a novel conceptual framework for understanding technology’s cumulative societal effects. Second, we identify five specific pathways through which AI systems possibly create technological externalities that accumulate beyond individual system impacts. Third, we propose governance mechanisms that address cumulative effects rather than individual system risks, contributing to broader discussions about democratic technology governance.

The central argument is that current technological governance approaches suffer from a fundamental assessment gap: they evaluate systems individually while missing collective impacts that may pose greater risks to human agency and social cohesion than any single application. By extending environmental health principles to cognitive and social domains, we can develop more comprehensive approaches to ensuring that technological development serves human flourishing rather than undermining it.

## 2 From environmental to cognitive-social protection

### 2.1 Expanding the analytical frame

Environmental health science offers a valuable framework for understanding technology’s potential long-term societal impacts. Just as Rachel Carson’s “Silent Spring” (1962) revealed how seemingly safe pesticide levels could accumulate into ecological disaster, we need frameworks for understanding how seemingly innocuous technological systems might accumulate into social and psychological disruption.

Consider lead exposure, once considered safe at “low levels.” We now know there is no safe threshold for lead in children’s blood (UNEP, 2025). Similarly, carbon emissions, individually negligible from a single vehicle, collectively transform global climate systems. The environmental health paradigm highlights three critical patterns that apply equally to technological exposure (Colborn et al., 1997):

First, cumulative effects exceed the sum of individual exposures. Just as multiple chemical exposures can interact synergistically, creating effects more severe than any single contaminant, the combined influence of multiple technological systems may amplify beyond their individual impacts.

Second, there is latency between exposure and manifestation. Environmental contamination often produces delayed effects, with decades passing before consequences fully emerge. Similarly, the cognitive and psychological effects of technological immersion may only become apparent after prolonged exposure.

Third, there is variable vulnerability across populations. Environmental toxins disproportionately affect developing bodies and marginalized communities (Colborn et al., 1997). Likewise, technological effects may particularly impact developing minds and vulnerable populations.

### 2.2 Conceptualizing AI as cognitive-social environmental contaminant

What constitutes “low-level technological exposure” in this framework? We define it as regular interaction with systems that subtly shape cognitive and emotional processes without crossing thresholds of perceived harm or risk. This includes recommendation algorithms that imperceptibly narrow information environments, predictive text systems that subtly shape language patterns, engagement-optimizing content feeds that modulate emotional states, convenience-enhancing AI assistants that gradually supplant skills, and personalization systems that incrementally reinforce existing beliefs.

None of these systems typically raise regulatory concerns under risk-based frameworks. Yet their collective presence creates a cognitive-social environment fundamentally different from the one human minds and societies evolved to navigate, a transformation comparable to how industrial technologies altered the physical environment our bodies evolved to inhabit.

This perspective may bring insights about how technologies become embedded in social practices and reshape human capabilities (Latour, 1992). However, while STS typically focuses on how technologies are socially constructed, our framework examines how they may be reconstructing human cognition and social connection through mechanisms that operate below the threshold of conscious awareness.

### 2.3 Related frameworks and differentiation

The proposition that digital technologies reshape cognition is not new. Several established research programs address overlapping dimensions, and our framework must be situated within that landscape.

The technostress literature (Tarafdar et al., 2015) documents how digital demands create acute and chronic stress responses. Our framework differs in focusing on effects operating below conscious awareness: the gradual reshaping of capacities through systems users perceive as benign. Where technostress asks how technology makes us feel, we ask how it changes what we are capable of feeling, thinking, and deciding.

Risko and Gilbert (2016) formalised cognitive offloading using external tools to reduce internal cognitive demands, and substantial work has examined how delegation affects memory (Sparrow et al., 2011), spatial cognition (Dahmani and Bohbot, 2020), and reasoning (Wilmer et al., 2017). Our framework extends this by embedding individual offloading within a population-level cumulative model that considers interactions across multiple domains simultaneously and translates findings into governance implications.

Yeung (2017) introduced “hypernudges”—Big Data-driven choice architectures that are networked, continuously updated, and pervasive. Our framework complements this regulatory analysis by examining the downstream psychological consequences of prolonged exposure to hypernudging environments.

Research on automation bias demonstrates that increasing automation reduces experienced agency (Berberian et al., 2012) and that environmental control manipulations modulate the prospective sense of agency, with individual vulnerability factors acting as moderators (Di Plinio et al., 2019). Our framework embeds these findings within a broader cumulative model, arguing that repeated micro-delegations across multiple systems may produce aggregate agency depletion.

Most directly adjacent, Di Plinio (2025) reviewed AI threats to agency and identity, advocating psychometric assessment tools and neurorights frameworks. Our key differentiation is analytical level: where Di Plinio focuses on individual psychological assessment, we introduce an environmental-population paradigm foregrounding cumulative effects across systems and proposing governance mechanisms drawn from environmental regulation.

The distinctive contribution of our framework is therefore not the identification of any single pathway, most have been documented in adjacent literatures, but three interrelated propositions: (1) individually documented effects interact and accumulate across domains in ways current frameworks cannot capture; (2) the appropriate governance response is cumulative impact monitoring analogous to environmental protection; and (3) the cognitive and social capacities affected constitute public goods whose degradation represents quantifiable externalities.

### 2.4 Operationalising cumulative exposure

If cumulative exposure is to serve as more than a metaphor, it requires operationalisation. Drawing on environmental health exposure science (WHO, 2020), we propose that cumulative AI exposure be characterised along five dimensions: frequency (how often one interacts with AI-mediated systems), duration (length of episodes and total lifetime exposure, including developmental windows), intensity (degree to which systems actively shape cognition through personalisation and engagement optimisation), diversity of systems (the number and variety of concurrent AI exposures, paralleling environmental mixture effects; Colborn et al., 1997), and developmental timing (life stage of exposure, given disproportionate vulnerability during cognitive development).

We further distinguish three cumulation models generating distinct testable predictions. In additive cumulation, total impact is the sum of individual exposures. In synergistic cumulation, interactions amplify effects beyond their additive sum—for example, algorithmic content curation combined with AI-mediated social interaction may reshape identity formation more profoundly than either alone. In threshold cumulation, effects remain negligible until a critical exposure level triggers nonlinear shifts.

Key moderators influencing individual vulnerability include baseline self-regulatory capacity (Diamond, 2013), social embeddedness, prior digital literacy, and socioeconomic context. These generate testable hypotheses about differential vulnerability and inform targeted intervention design. This operationalisation does not resolve measurement challenges, but transforms cumulative exposure from a rhetorical device into a structured analytical framework with identifiable dimensions and testable interaction models.

## 3 Five pathways of cognitive-social contamination

Two clarifications regarding the five pathways are necessary. First, while presented as analytically distinct, they overlap and interact causally. Attention erosion may exacerbate emotional dependency; decision-making dependency may feed back into identity fragmentation. These feedback loops are central to the cumulative-exposure thesis: the interaction among pathways may produce aggregate effects exceeding the sum of individual impacts. Second, the pathways are not exhaustive; they represent domains where current evidence is most suggestive.

We also note a definitional boundary. While our pathways draw on broader digital technology research, our argument is that AI (systems using machine learning to optimise outputs based on user data) represents a qualitative intensification of these dynamics. Where we draw on non-AI-specific evidence, we note where extrapolation requires further validation. Each pathway below distinguishes: (a) empirical regularities, (b) plausible mechanisms, and (c) speculative cumulative hypotheses with testable predictions.

### 3.1 Attention erosion

#### 3.1.1 Empirical regularities

Heavy media multitaskers show greater susceptibility to interference, though meta-analytic pooled effect sizes are small (Parry and le Roux, 2021). Wilmer et al. (2017) found suggestive but largely correlational associations between smartphone habits and attentional difficulties. Ward et al. (2017) reported smartphone mere presence reduces cognitive capacity, though replication has produced mixed results (Ruiz Pardo and Minda, 2022). Collectively, evidence indicates a pattern of associations, though predominantly correlational and small to moderate in effect size (Delgado et al., 2018).

#### 3.1.2 Plausible mechanisms

The cognitive offloading framework (Risko and Gilbert, 2016) suggests that when external systems consistently manage information filtering, internal attentional processes may undergo functional reorganisation (Clark, 2015). AI-driven content optimisation intensifies this through continuous real-time personalisation, creating feedback loops between attention patterns and algorithmic content selection that may progressively narrow attentional repertoires.

#### 3.1.3 Speculative cumulative hypothesis

We hypothesise that prolonged population-wide exposure to AI-optimised environments may produce measurable shifts in sustained attention distributions—most pronounced in individuals whose exposure began during developmental periods and interacting synergistically with diversity of system exposure. If sustained, such shifts may have consequences for democratic governance, which depends on citizens’ capacity to engage with complex policy arguments over extended periods. A population whose attentional baseline has shifted toward fragmented processing may find deliberative demands increasingly difficult to meet—a subtle form of democratic capacity erosion that no individual system produces but that the cumulative environment may generate.

Testable predictions: This hypothesis would be weakened if longitudinal studies show no attentional changes beyond pre-existing traits, if populations with different exposure levels show no distributional differences, or if changes prove fully reversible.

### 3.2 Emotional dependency

#### 3.2.1 Empirical regularities

Odgers and Jensen (2020) caution that large-scale preregistered studies report small associations between digital technology use and adolescent wellbeing, unlikely to be of clinical significance in isolation. Nevertheless, algorithmic content curation modulates mood states (Kramer et al., 2014), and emerging research on AI companion use suggests patterns of emotional reliance (Pentina et al., 2023).

#### 3.2.2 Plausible mechanisms

Two mechanisms are proposed: emotional outsourcing meaning reliance on algorithmically mediated stimulation rather than developing internal regulation capacities; and emotional calibration drift—prolonged exposure to amplified emotional content shifts baseline expectations, making naturally occurring experiences relatively less satisfying.

#### 3.2.3 Speculative cumulative hypothesis

Cumulative exposure may gradually erode internal emotional regulation, producing increased reactivity without digital stimulation and difficulty with self-directed regulation in non-digital contexts. This engages fundamental questions about agency: if agency requires emotional self-regulation then gradual outsourcing of this capacity may undermine agency at its affective foundation (Borgmann, 1984), not through restricting choices but through atrophy of capacities that make autonomous choice meaningful.

Testable predictions: Weakened if emotional regulation shows no decline in heavy users over longitudinal observation, or if changes are fully attributable to pre-existing differences.

### 3.3 Social connection alteration

#### 3.3.1 Empirical regularities

AI-mediated communication strips multimodal cues triggering bonding responses (Sætra, 2020; Pugh, 2023); algorithmic filtering curates encounters by engagement criteria rather than relationship-building criteria. Recent research shows algorithmic personalization impacts social connectedness (Taylor and Chen, 2024; Wang et al., 2022).

#### 3.3.2 Plausible mechanisms

Proposed mechanisms include: social signal attenuation when AI-mediated interaction reduces the neurochemical and nonverbal signals that underlie empathy and social bonding; and algorithmic relationship filtering when platforms prioritize engagement metrics over authentic relationship development.

#### 3.3.3 Speculative cumulative hypothesis

We hypothesise cumulative reliance on AI-mediated social interaction may reduce empathic accuracy and alter attachment patterns, with effects scaling with diversity of mediating systems. This technological mediation represents what Borgmann (1984) identified as the “device paradigm,” where technologies promise to deliver social goods while transforming their very nature.

Testable predictions: Weakened if empathy measures show no decline with high vs. low AI-mediated interaction, controlling for total social interaction quantity.

### 3.4 Decision-making dependency

#### 3.4.1 Empirical regularities

This pathway has the strongest empirical support. GPS use associates with poorer spatial memory (Dahmani and Bohbot, 2020; Gramann et al., 2017); knowing information is accessible reduces encoding effort (Sparrow et al., 2011); increasing automation reduces sense of agency (Berberian et al., 2012), with individual vulnerability factors moderating the effect (Di Plinio et al., 2019).

#### 3.4.2 Plausible mechanisms

Cognitive offloading creates a self-reinforcing dependency: as unassisted decision-making becomes more effortful through disuse, the case for further delegation strengthens.

#### 3.4.3 Speculative cumulative hypothesis

We hypothesise cumulative delegation across multiple systems may produce generalised decision-making dependency (diminished confidence in unassisted judgment and heightened anxiety without algorithmic mediation) with consequences for the independent judgment democratic participation requires. If citizens increasingly rely on algorithmic mediation for daily decisions, their capacity for the independent judgment that democracy requires may be gradually undermined.

Testable predictions: Weakened if decision-making competence in non-delegated domains remains unaffected by heavy delegation elsewhere.

### 3.5 Identity formation

#### 3.5.1 Empirical regularities

Personalisation algorithms impact self-perception (Taylor and Chen, 2024), with correlations between social media use and identity distress. This effect appears particularly pronounced in developmental stages where identity formation occurs most actively.

#### 3.5.2 Plausible mechanisms

Proposed mechanisms include: algorithmic identity capture—personalisation narrows the user’s sense of possibility to what the algorithm predicts; and performative self-optimisation—identity formation shifts toward external, algorithmically-optimised performance (Turkle, 2015).

#### 3.5.3 Speculative cumulative hypothesis

We hypothesise prolonged exposure may reduce capacity for self-directed identity exploration. Following Taylor (1992), the concern is not that AI presents a “false” self but that algorithmic recognition (optimised for engagement) may progressively displace the broader frameworks of meaning through which authentic identity (the ongoing capacity to define oneself through meaningful frameworks of value and commitment rather than externally imposed categories) has historically been constituted. Not forbidding authenticity but making it increasingly effortful.

Testable predictions: Weakened if identity coherence shows no association with algorithmic personalisation exposure.

## 4 The governance gap: why current technological governance falls short

Current technological governance approaches typically assess systems individually, asking whether a specific application creates substantial risk. This resembles early environmental regulation that evaluated individual chemicals in isolation, missing their combined impact. Just as individually “safe” levels of multiple toxins can collectively exceed safety thresholds, AI systems deemed “low-risk” individually may collectively transform cognitive and social environments beyond resilience thresholds.

This mirrors findings in environmental health research where combinations of pollutants at concentrations below regulatory limits can produce unexpected health impacts (UNEP, 2025). Environmental contaminants often interact in complex ways that regulatory frameworks fail to capture, with additive or synergistic effects that only become apparent through cumulative exposure.

Consider recommendation systems: Any single recommendation engine likely falls below risk thresholds in regulatory frameworks. Yet when dozens of such systems collectively shape information exposure across multiple platforms daily, they potentially create information environments that systematically undermine epistemic agency and democratic deliberation. This cumulative effect remains largely unaddressed in system-by-system assessment approaches.

This governance gap reflects what Scott (1998) identified as the limitations of “seeing like a state” regulatory frameworks that break complex socio-technical systems into discrete, manageable components may miss the emergent properties that arise from their interaction.

Environmental regulation evolved partly in response to the concept of externalities costs imposed on parties who did not choose to incur them (Colborn et al., 1997). AI systems similarly produce cognitive and social externalities that remain largely unaccounted for in governance frameworks.

When an AI system optimizes for user engagement, corporate profit, or operational efficiency, it potentially creates technological externalities: attention fragmentation, decision dependency, emotional modulation, and social disconnection. These effects do not appear on corporate balance sheets or regulatory risk assessments, yet they represent real costs to cognitive sovereignty, social cohesion, and democratic capacity that accumulate over time.

This externality gap grows as AI systems increasingly mediate everyday experiences while optimisation objectives remain misaligned with human flourishing and democratic governance (Zuboff, 2019). Without mechanisms to account for these externalities, market forces alone will likely increase rather than mitigate cumulative technological impacts on society.

Environmental regulation advanced significantly with improved detection methods that could measure previously imperceptible contamination levels. Similarly, addressing cumulative technological effects requires developing metrics and measurement approaches for cognitive, emotional, and social impacts that currently remain largely unmeasured. How do we quantify the erosion of decision making independence? What constitutes a “dangerous level” of attention fragmentation? How can we measure the degree of algorithmic influence on identity formation or social bonding capacity? How do we assess changes in empathic accuracy or democratic deliberation quality? Without established metrics and thresholds, governance frameworks struggle to incorporate these dimensions into technological assessment models (Zuboff, 2019). This measurement challenge represents both a technical obstacle and a fundamental conceptual hurdle for effective governance of cumulative technological effects. Developing appropriate metrics requires not just new measurement tools but new conceptual frameworks for understanding human-technology ecologies that span cognitive, emotional, and social domains.

## 5 Proposed governance innovations for democratic technology development

### 5.1 From individual to cumulative technological assessment

Environmental protection combines individual health practices with systemic regulation personal choices to avoid contaminants alongside policies that limit emissions. Similarly, addressing AI’s cumulative effects requires both individual technological hygiene practices and systemic governance innovations that ensure technology serves democratic purposes.

Individual practices like attention management, emotional awareness, intentional technology use, maintaining authentic social connections, resemble personal health measures like filtering drinking water or reducing exposure to environmental toxins. These practices provide important protection but remain insufficient without complementary systemic measures that address root causes and ensure technological development serves human flourishing.

Effective technological governance must move beyond risk-based assessment of individual systems to consider cumulative effects across cognitive, emotional, and social environments. This might include holistic technological impact assessments that evaluate how systems collectively shape attention, emotional regulation, social connection, decision-making, and identity formation; algorithmic impact labels that disclose how systems influence psycho-emotional patterns, similar to environmental impact disclosures; cumulative exposure monitoring across platforms and applications, analogous to environmental monitoring networks; developmental preservation zones that limit technological optimisation pressures during critical developmental periods; and social authenticity standards that prevent manipulation of social and emotional responses through design patterns that prioritize engagement over human wellbeing. Current algorithmic auditing exemplified by the SMACTR framework (Raji et al., 2020) focuses on individual system harms. We propose extending this through: (i) cross-system exposure mapping at the SMACTR scoping stage, requiring developers to consider how their system interacts with the broader AI ecosystem users already navigate; (ii) longitudinal impact modeling, specifying expected interaction patterns and cumulative trajectories; and (iii) cumulative threshold analysis, assessing whether a system’s contribution might push populations past critical exposure levels. Given the adaptive nature of AI systems (Yeung, 2017), these assessments should function as living documents with regular review cycles, not one-time pre-deployment gates.

### 5.2 Cognitive-social environmental monitoring

We propose initial population-level indicators: sustained attention capacity (measured via standardised tasks such as the SART; Robertson et al., 1997), emotional self-regulation (Difficulties in Emotion Regulation Scale; Gratz and Roemer, 2004), social connection quality (integrated into existing surveys such as the European Social Survey), decision-making independence (experimental tasks plus self-report algorithmic reliance measures), and identity coherence (Sense of Coherence scale; Antonovsky, 1987). These are designed to be administrable through existing infrastructure like PISA assessments, European Social Survey, national health surveys, minimising institutional overhead while generating longitudinal data.

### 5.3 Synthetic population modeling as predictive governance tool

Unlike environmental contaminants, where decades of epidemiological data now inform regulation, we cannot wait for longitudinal studies to reveal technology’s long-term cognitive and social effects. The pace of technological change and deployment far exceeds traditional research timelines. Instead, we should look for alternative methods. One could consider synthetic population modeling as an evidence-based predictive approach for democratic technology governance. Synthetic populations computer generated models of individuals with realistic attributes can simulate how technological exposure might influence cognitive, emotional, and social outcomes over extended timeframes. These models allow testing evidence-based hypotheses about cumulative effects without waiting decades for real-world data, potentially identifying concerning patterns before they become entrenched in actual populations.

This approach builds on established methods in digital twin technology, where virtual replicas of physical entities enable predictive simulation. Recent advances in AI-enabled digital twins allow for high-fidelity modeling of complex systems, predicting behaviors and outcomes under various conditions (Taylor et al., 2023; Biller and Biller, 2023).

For example, synthetic populations could model how various levels of social media algorithm exposure might influence democratic participation patterns in different demographic groups over a simulated decade. These simulations could incorporate feedback loops (e.g., increased polarization leading to decreased civic engagement) and test potential interventions (e.g., reduced algorithmic personalization or enhanced media literacy programs).

This approach resembles how climate scientists use computational models to forecast potential outcomes based on current trends and mechanisms, allowing for evidence-based policy development without requiring catastrophic events to first occur. While imperfect, such models provide actionable insights when combined with existing research, theoretical frameworks, and emerging evidence potentially avoiding the decades-long delay between early warning signs and effective regulation that characterised many environmental contaminants.

### 5.4 Cognitive-social ecosystem services valuation

Just as clean water and unpolluted air provide essential ecosystem services that underpin physical health and economic prosperity, our cognitive and social capacities constitute “mental ecosystem services” that support human flourishing and democratic governance. These cognitive-social commons, including sustained attention, emotional authenticity, genuine social connection, independent decision making, and coherent identity formation represent resources as vital as any natural capital, yet they remain largely unprotected from technological disruption.

The breakthrough 1997 study that valued global ecosystem services at $33 trillion annually (exceeding the global GNP of $18 trillion) transformed environmental protection by demonstrating nature’s economic significance (Costanza et al., 1997). Similarly, we must quantify our cognitive-social ecosystem services’ contribution to human flourishing and democratic governance. Their true social and economic value would likely approach several percentage points of global GDP, encompassing everything from the capacity for democratic deliberation to the social trust that enables economic cooperation.

Recognizing these cognitive-social ecosystem services not as individual luxuries but as quantifiable public goods essential for democratic society will drive the governance frameworks needed to preserve them from technological degradation. This approach aligns with broader movements toward recognizing the social value of commons that cannot be reduced to market transactions (Ostrom, 1990).

### 5.5 Validation strategy and phased implementation

We propose phased implementation: Phase 1 (Years 1–3)—establish baseline monitoring in pilot populations with varying AI exposure, testing whether cumulative exposure predicts cognitive-social variance beyond demographic confounders; Phase 2 (Years 2–4)—pilot cumulative impact assessment with AI developers; Phase 3 (Years 3–5)—integrate validated metrics into existing survey infrastructure; Phase 4 (Years 5+)—review and refine. This design is responsive to findings: if Phase 1 reveals no significant associations, the case for intensive governance diminishes accordingly.

### 5.6 Regulation-by-design for adaptive systems

For AI systems that continuously evolve (Yeung, 2017), we propose: effect boundary specification (developers define the range of algorithmic parameter variation), adaptation monitoring (parameter evolution logs accessible to auditors), and circuit-breaker mechanisms (automated review triggers when impact thresholds are approached). These requirements align with the EU AI Act’s (European Parliament, 2024) risk-tiered approach while extending it to capture cumulative dynamics.

## 6 Implications for technology-society research and practice

This environmental paradigm offers several theoretical contributions to technology-society literature. First, it shifts focus from individual technological risks to cumulative exposure effects, revealing previously invisible governance gaps that affect democratic capacity. Second, it provides a conceptual bridge between environmental health science and technology governance, suggesting productive cross-disciplinary collaboration for understanding technology’s societal impacts (Zuboff, 2019). Third, it operationalizes concepts like “technological externalities” and “cognitive-social ecosystem services” that could inform broader technology governance frameworks focused on human flourishing rather than mere efficiency.

The framework also suggests new research directions for technology studies. Empirical studies could test the proposed pathways through longitudinal analysis of technological exposure and psychological outcomes. Computational models could explore cumulative effect mechanisms through agent-based simulation of technology-society interactions. Policy research could evaluate the effectiveness of proposed governance innovations through natural experiments and quasi-experimental designs that examine different technological governance approaches.

For policymakers, this framework suggests several immediate applications that could strengthen democratic governance of technology. Technological impact assessments could incorporate cumulative effect analysis alongside individual system evaluation, ensuring that governance decisions consider broader societal implications. Regulatory sandboxes could test cumulative monitoring approaches at smaller scales before broader implementation, allowing democratic experimentation with new governance models. International cooperation could develop shared standards for measuring and governing technological externalities, preventing a “race to the bottom” in technology governance.

For technology developers, the framework implies design principles that consider cumulative impacts alongside individual system performance and business objectives. This might include attention-preserving interface design that supports rather than fragments human cognition, emotional authenticity standards that prioritize human wellbeing over engagement metrics, and algorithmic transparency measures that enable user agency rather than technological dependency.

For researchers, the framework suggests methodological innovations including synthetic population modeling for predicting technological impacts, longitudinal exposure studies that track technology’s effects on human development, and cross-platform impact assessment that examines how multiple technological systems interact to shape human experience. It also implies new metrics development for cognitive, emotional, and social impacts that current approaches cannot capture but that may be essential for preserving human agency in a technological society.

This theoretical framework has several limitations that future research should address. First, the environmental analogy, while illuminating, may not capture all aspects of technology’s societal impacts. Unlike chemical exposure, technological interaction involves conscious agency and social construction, creating different dynamics than passive contamination. Future research should explore how human agency and technological influence interact in complex ways that neither technological determinism nor social constructivism alone can explain.

Second, the proposed pathways require empirical validation through controlled studies that can establish causal rather than correlational relationships. The GPS navigation research provides a compelling example of how technology can reshape cognitive capacities, but similar evidence is needed across other domains to validate the broader framework.

Third, the governance innovations proposed here require testing through pilot programs and policy experiments before broader implementation. The synthetic population modeling approach, in particular, needs validation through comparison with real-world outcomes and democratic input into the values and assumptions built into such models.

Future research should prioritize developing measurement tools for the proposed pathways, conducting longitudinal studies of cumulative technological exposure across different populations, and testing governance innovations through controlled policy experiments that examine their effectiveness and democratic legitimacy. Cross-disciplinary collaboration between technology researchers, environmental health scientists, social scientists, and policy scholars will be essential for advancing this framework in ways that strengthen rather than undermine democratic governance.

## 7 Conclusion

We stand at a moment comparable to the early days of environmental awareness, when the first evidence of widespread contamination emerged but comprehensive protection frameworks had not yet developed (Carson, 1962). The human cognitive and social environment now faces similar challenges, but subtle pervasive alterations from technologies deployed faster than their democratic implications can be fully understood or governed.

The environmental analogy offers both caution and hope for democratic technology governance. Environmental degradation continued for decades before effective protection emerged, with substantial and sometimes irreversible damage occurring in the interim (Carson, 1962; Colborn et al., 1997). Yet environmental protection eventually developed into sophisticated governance systems that significantly reduced many forms of contamination while allowing continued technological advancement guided by ecological principles.

By recognizing AI’s potential cumulative effects on human agency and social cohesion now, and by developing governance approaches that address the quality of our cognitive, emotional, and social environments alongside individual system risks we have the opportunity to practice democratic technology stewardship. This means preserving the cognitive-social commons that underlie human flourishing and democratic governance, ensuring that technological development serves rather than undermines the capacity for meaningful choice and authentic connection.

Just as clean water and air are essential for physical health, uncontaminated attention, emotional authenticity, genuine social connection, independent decision-making, and coherent identity formation are essential for psychological wellbeing and democratic participation. By extending environmental protection paradigms to these domains of human experience, we can develop governance approaches that preserve these fundamental capacities while still embracing technology’s potential benefits for human flourishing.

The stakes could not be higher for democratic society. If we fail to address these cumulative impacts, we risk a future where human agency and social cohesion gradually erode not through sudden technological disruption, but through the silent accumulation of cognitive and social effects that undermine the very foundations of democratic self-governance. The time to develop comprehensive protection for our cognitive-social environment is now, before such effects become both severe and irreversible.

## Data Availability

The original contributions presented in the study are included in the article/supplementary material, further inquiries can be directed to the corresponding author.
